# Supplementary material for: Identification of NCAPG as an Essential Gene for Neuroblastoma Employing CRISPR-Cas9 Screening Database and Experimental Verification
Source: Int J Mol Sci. 2023 Oct 6;24(19):14946. doi: 10.3390/ijms241914946 (PMC10573393; doi:10.3390/ijms241914946)
Supplement: Supplementary file 1 [file ijms-24-14946-s001.zip › Figure S1.pdf]

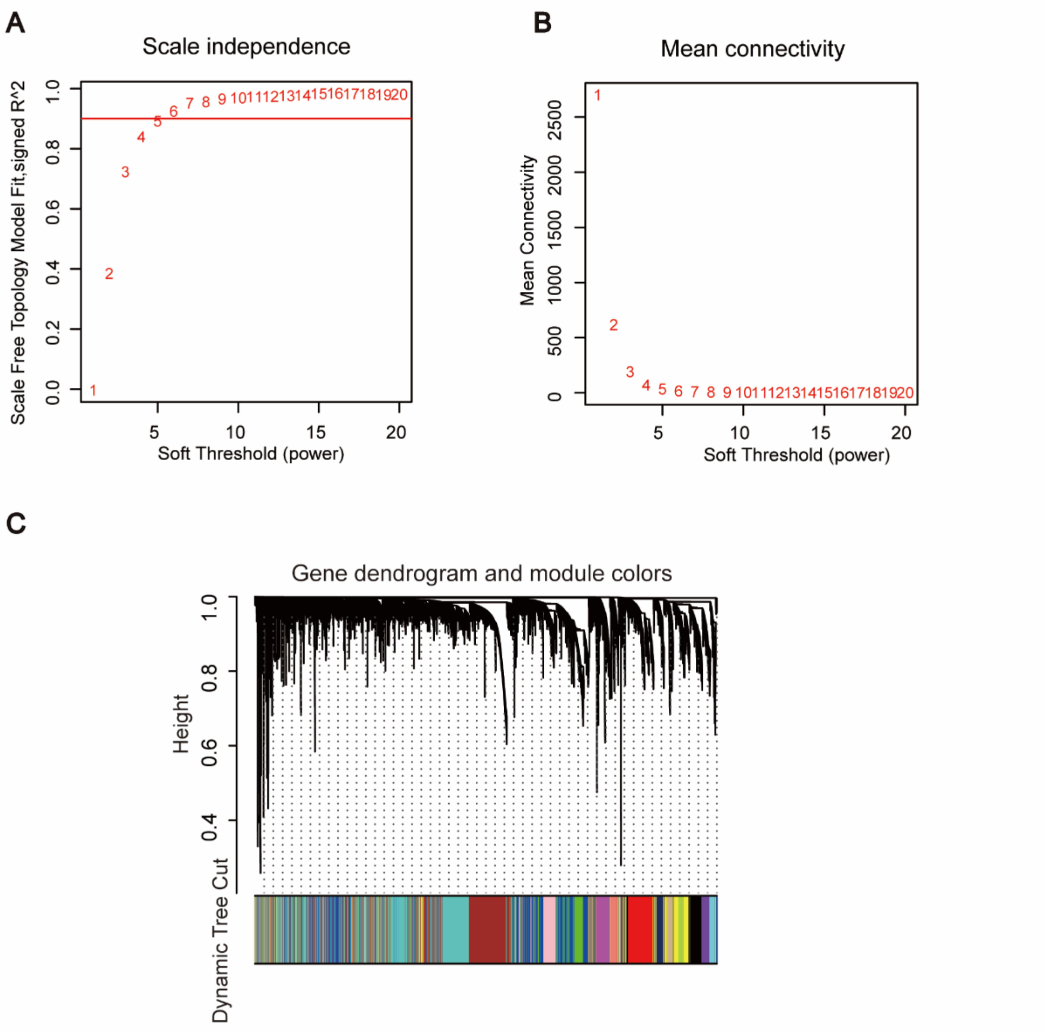

Figure S1. Analysis performed by WGCNA using data from GSE49710. (**A, B)** Scale-free fit index analyses for various soft threshold levels, the numbers in the picture represent the corresponding soft threshold power. (**C**) mRNA co-expression network modules, the colors at the bottom of the image represent different modules.
