# Supplementary material for: Identification of NCAPG as an Essential Gene for Neuroblastoma Employing CRISPR-Cas9 Screening Database and Experimental Verification
Source: Int J Mol Sci. 2023 Oct 6;24(19):14946. doi: 10.3390/ijms241914946 (PMC10573393; doi:10.3390/ijms241914946)
Supplement: Supplementary file 1 [file ijms-24-14946-s001.zip › Figure S2.pdf]

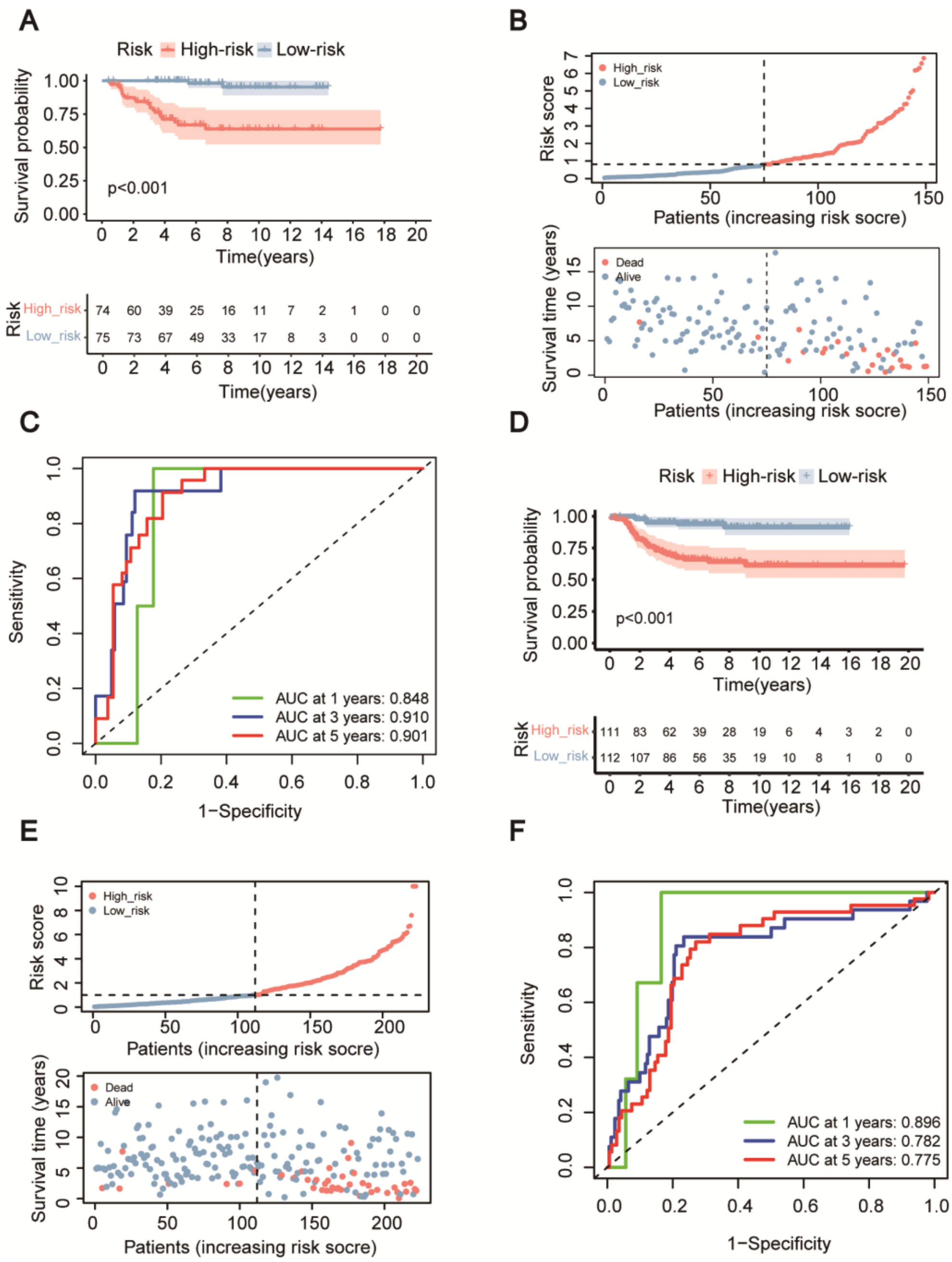

Figure S2. Validation of the neuroblastoma prognostic signature. (**A**) Plot showing the Kaplan-Meier distribution for the internal validation cohort. (**B**) Each patient in the internal validation cohort's risk score and survival status distributions were displayed. (**C**) ROC curves for internal validation cohort survival prediction. (**D**) Plot showing the Kaplan-Meier distribution for the external validation cohort. (**E**) Each patient in the external validation

cohort's risk score and survival status distributions were displayed. (**F**) ROC curves for external validation cohort survival prediction.
