# Supplementary material for: Identification of NCAPG as an Essential Gene for Neuroblastoma Employing CRISPR-Cas9 Screening Database and Experimental Verification
Source: Int J Mol Sci. 2023 Oct 6;24(19):14946. doi: 10.3390/ijms241914946 (PMC10573393; doi:10.3390/ijms241914946)
Supplement: Supplementary file 1 [file ijms-24-14946-s001.zip › Figure S3.pdf]

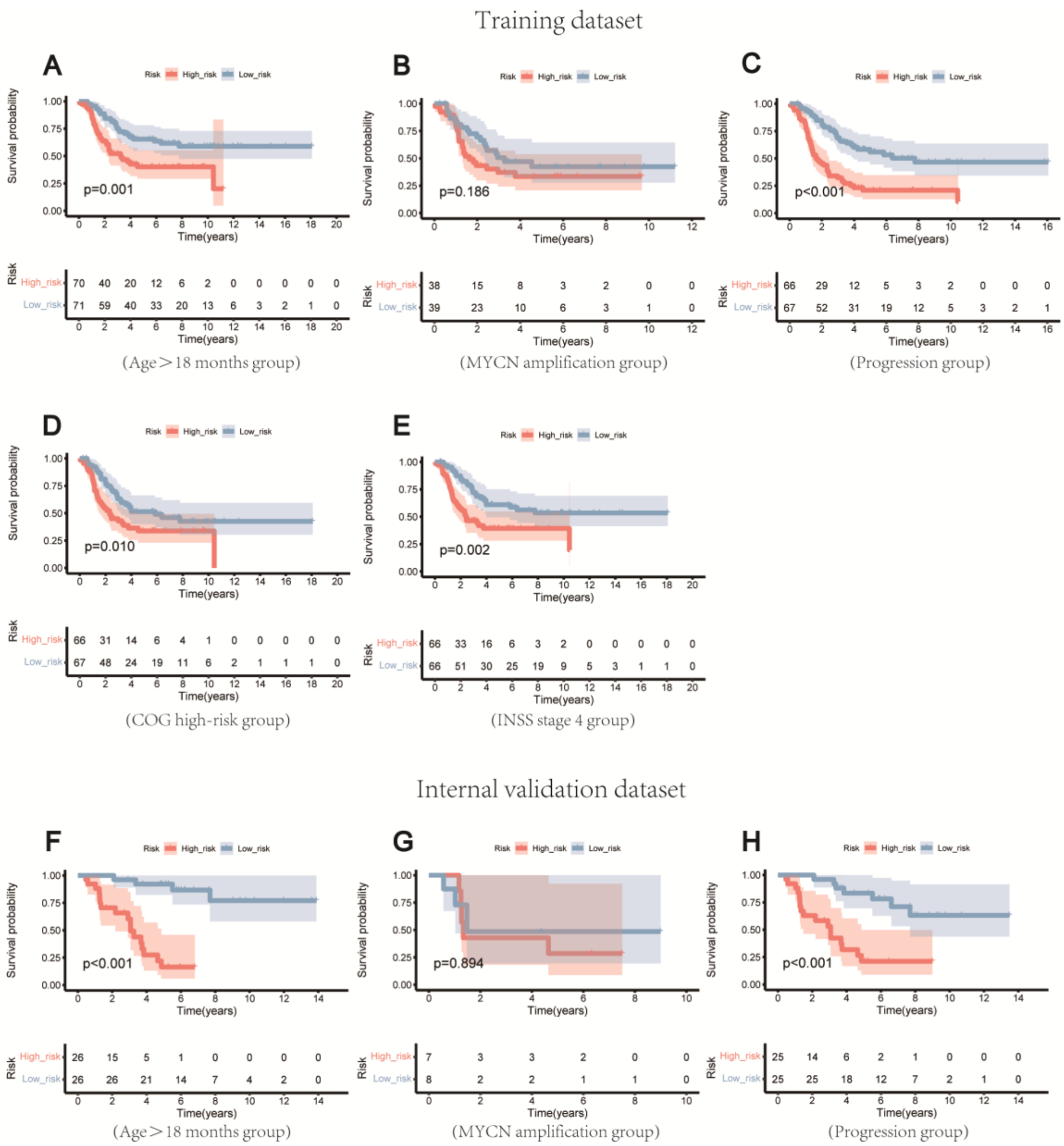
Training dataset
A
B
C
D
E
Risk
High_risk
Low_risk
Survival probability
Time(years)
p=0.001
p=0.186
p<0.001
p=0.010
p=0.002
(Age＞18 months group)
(MYCN amplification group)
(Progression group)
(COG high-risk group)
(INSS stage 4 group)
Internal validation dataset
F
G
H
p<0.001
p=0.894
p<0.001
(Age＞18 months group)
(MYCN amplification group)
(Progression group)

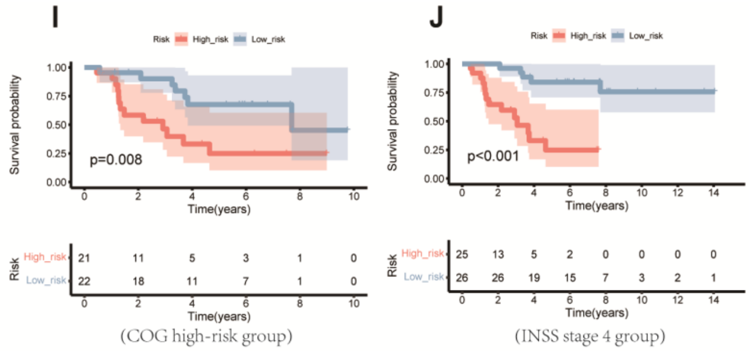

External validation dataset (E-MTAB-8248)

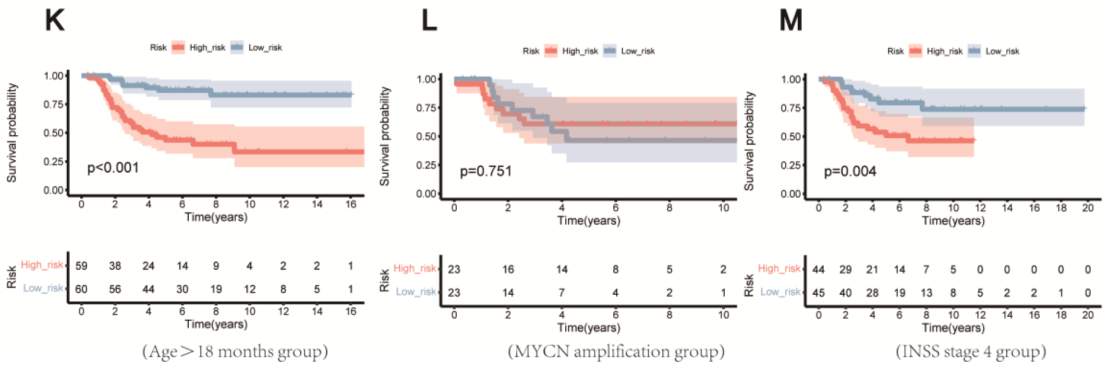

Figure S3. The prognostic value of the risk signature in samples with unfavorable characteristics. (A-E) The Kaplan–Meier curve of the risk signature in patients with age > 18 months (A), MYCN amplification (B), progression (C), COG high-risk group (D), and INSS stage 4 (E), in the training dataset. (F-J) The Kaplan–Meier curve of the risk signature in patients with age > 18 months (F), MYCN amplification (G), progression (H), COG high-risk group (I), and INSS stage 4 (J), in the internal validation dataset. (K-M) The Kaplan–Meier curve of the risk signature in patients with age > 18 months (K), MYCN amplification (L), and INSS stage 4 (M), in the external validation dataset.
