# Supplementary material for: Identification of NCAPG as an Essential Gene for Neuroblastoma Employing CRISPR-Cas9 Screening Database and Experimental Verification
Source: Int J Mol Sci. 2023 Oct 6;24(19):14946. doi: 10.3390/ijms241914946 (PMC10573393; doi:10.3390/ijms241914946)
Supplement: Supplementary file 1 [file ijms-24-14946-s001.zip › Figure S4.pdf]

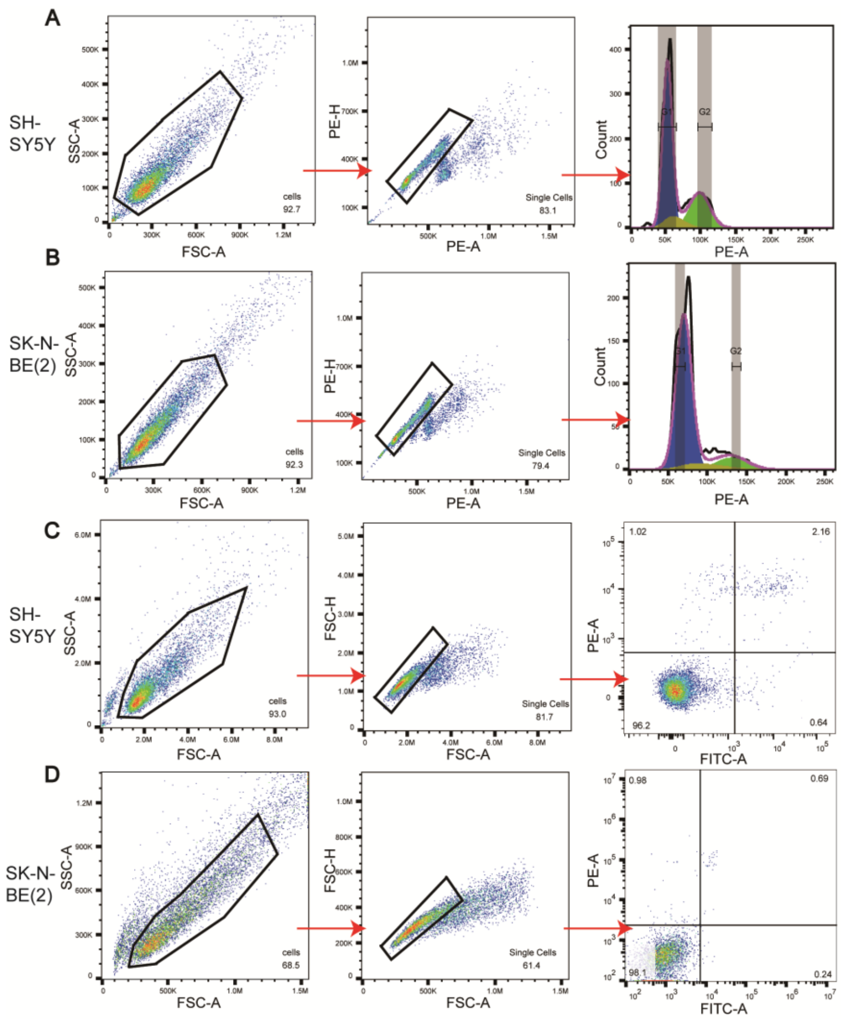

Figure S4. The flow cytometry gating strategy. (A-B) Flow cytometry gating strategy for cell cycle in SH-SY5Y and SK-N-BE (2), the blue peak represents G0/G1 phase cells; the yellow peak represents S phase cells; the green peak represents G2/M phase cells. (C-D) Flow cytometry gating strategy for apoptosis in SH-SY5Y and SK-N-BE (2).
