# Supplementary material for: Identification of NCAPG as an Essential Gene for Neuroblastoma Employing CRISPR-Cas9 Screening Database and Experimental Verification
Source: Int J Mol Sci. 2023 Oct 6;24(19):14946. doi: 10.3390/ijms241914946 (PMC10573393; doi:10.3390/ijms241914946)
Supplement: Supplementary file 1 [file ijms-24-14946-s001.zip › Table S3.pdf]

Table S3. clinical characteristics of the neuroblastoma patients

Characteristic	Patient number (%)
Total	40
Gender	
Male	16(40.0)
Female	24(60.0)
Age(months)	
<18	11(27.5)
≥18	29(72.5)
INSS stage	
1	13(32.5)
2	3(7.5)
3	4(10.0)
4	18(45.0)
4s	2(5.0)
COG risk	
LR	16(40.0)
IR	2(5.0)
HR	22(55.0)
Bone marrow metastasis	
No	24(60.0)
Yes	16(40.0)
Distant metastasis	
No	20(50.0)
Yes	20(50.0)
Histological type	
Ganglioneuroblastoma	12(30.0)
Neuroblastoma	28(70.0)
MYCN amplification	
No	28(70.0)
Yes	12(30.0)
Relapse	
No	22(55.0)
Yes	18(45.0)
Follow-up	
Alive	27(67.5)
Dead	13(32.5)
